# Importance of personal development for a diverse workforce in thoracic surgery: the art of Curriculum Vitae building and interviewing

**DOI:** 10.1093/icvts/ivad144

**Published:** 2023-09-02

**Authors:** Cecilia Pompili, Isabelle Opitz, Emily Elswick, Nuria Novoa, Molly Cabalo, Beatrice Beck Shimmer, Peyman Sardari Nia, Brendon Stiles

**Affiliations:** St Bartholomew's Hospital, London, UK; University of Zurich, Zurich, Switzerland; Medtronic, Minneapolis, MN, USA; University Hospital Puerta de Hierro—Majadahonda, Madrid, Spain; Medtronic, Minneapolis, MN, USA; University of Zurich, Zurich, Switzerland; Maastricht University Medical Center, Maastricht, Netherlands; Albert Einstein College of Medicine and Montefiore Medical Center, New York, NY, USA

**Keywords:** Personal development, Thoracic surgery, Academic surgery, Diversity & Inclusion

Different surveys [1, 2] and reports [3, 4] have now demonstrated that thoracic surgery is not immune to gender bias, discrimination in training programs and in promotions to leadership positions.

Although female students are better represented in medical school and residency programs across Europe, there remains a gross under-representation of women in leadership positions, especially in academia where gender biases have been reported, even in letters of recommendations [5].

Several behavioural theories have been raised recently to underline the common obstacles women have to face regardless of their rank, cultural and institutional background. From the imposter [6] to the tall poppy syndrome [7], raising awareness and developing speak-up strategies have been demonstrated to be some of the most powerful ways to address perceived inequalities in the modern working environment.

The European Society of Thoracic Surgery (ESTS) has recently established the Women in General Thoracic Surgery Committee to develop initiatives to help and support female surgeons around the world. To face the everyday challenges that arise due to gender bias, responders to a cross-cultural survey [1] identified that initiatives are lacking in several countries that would help amplify their strengths.

One of the most important ones is highlighted by the lack of formal leadership and personal development (PD) courses at national or institutional levels. For this reason, the ESTS WGTS Committee has organized an academy where PD and leadership are among the topics covered, focusing on experience and examples from academic and industry leaders. Preparing a Curriculum Vitae (CV), developing best strategies for an interview and understanding institutional and societal drivers to support a diverse workforce may help future generations of minority and underrepresented groups develop a career in CT surgery.

## BUILDING YOUR CURRICULUM VITAE IN ACADEMIA

*Prof. Dr. B. Beck Schimmer*

*Vice President Medicine*

*University of Zurich-CH*

Building an academic career is not something that happens overnight. It takes dedication and hard work and requires a strategic plan with well-defined goals and a clear focus. But at the same time, you need to remain flexible just in case plan A falls apart. For a clinician-scientist, it is important to establish a career based on the cornerstones of clinical experience, research, and leadership, not forgetting the importance of networking. A mix of all this, together with good mentoring, can pave the way towards a successful career. Here is a more detailed overview of some of the important elements.

To build a CV in academia, you should aim to fill your backpack with experience. As a clinician, you of course need solid ‘clinical experience’, ideally with specialization in a particular field. Just as important is establishing yourself on the ‘research track’, by completing a doctoral thesis, attracting third-party funding, publishing important findings, developing, and establishing your own research group and carving out a niche in the scientific landscape. One of the overall goals is to become independent, especially in science.

Another key competence is ‘leadership’, which is often neglected. It is crucial to develop leadership skills by training in this area, as in an academic career you will have to lead and manage diverse teams in different environments. No one is born a master and there are many ways to develop in this field.

Participation in a ‘mentoring program’ is very useful at different stages of your career. Mentoring programs help you identify and achieve career goals, build confidence and give you the opportunity to share knowledge, experience and expertise in peer groups. In addition, mentoring programs can provide emotional support and guidance to help you manage far-reaching decisions or situations. Mentoring supports personal growth and development.

‘Networking’ is essential within the global science community. Exchange with other researchers provides access to new ideas and trends and can lead to fruitful collaborations or even interesting job offers.

Overall, building an academic career requires hard work, dedication and passion. You are embarking on a complex path. Stay focused on your goals, seek out opportunities to grow and develop your skills and don’t be afraid to take risks. Be curious! Alongside listing your professional and academic achievements, your CV should reflect this spirit, demonstrating your enthusiasm for what you do and for what you want to achieve in the future.

## ESSENTIALS FOR PREPARING INTERVIEWING

*Molly Cabalo,*

*Medtronic Talent Acquisition Consultant-US*

There are several key items you will want to consider when preparing to start your interview process including updating your CV, cover letter and profile. Next, you will want to prepare and do your research, know what to expect and prepare for in the interview and then properly follow up.

Your CV should be compelling, grabbing the reader’s attention so they want to learn more about you. Your most recent experience should be first. Include quantitative details that include action verbs that are clear and easy to review. Provide details that showcase the level of responsibility you had in your roles, avoiding flowery words and paragraphs. Adding a customized cover letter is a good idea. Take this opportunity to share how the skills you have make you a great match for the opportunity. Keep this to 1 page! The cover letter will rarely get you the job but do not let it be the reason you lose the job. For both the CV and the cover letter, triple check your spelling and grammar, and asking a colleague or friend to review is always a great idea!

A LinkedIn profile is an important part of your search. Even if you do not think you need it, there is a strong change those interviewing you will be looking at your profile. Include all of your training, memberships, recognition, volunteer work, languages spoken, etc.

You will want to do your research on the role and the organization. Network with others you know in the organization. Know that everything you are doing now is part of an interview process, every person you come across can positively or negatively impact your future interviews. Once you have secured the interview, it is time to prepare. Gather information about the role, the facility and the team, leveraging your internal network. Learn your interviewer’s titles, background, etc. Anticipate questions, prepare responses, and know your story and highlights. Prepare to answer with the STAR approach (explain the Situation/Task, your Action and the Results).

On the day of the interview you will want to dress professionally. If you are not sure, wear a suit. Bring extra copies of your resume on quality paper, along with a reference list and a portfolio with documentation of achievements.

During the interview, greet with a firm handshake, make eye contact and smile. Ask engaging questions while showing your passion for the opportunity. Come to the interview prepared with questions about the facility, the team, important criteria to be successful, growth opportunities and work environment. You will want to invite the interviewer to ask you any additional questions about your experience, knowledge and skills. The first interview may not be the time to push on salary, hours, vacation, free time, etc. If you have a Zoom interview scheduled, be sure to choose a professional background in a well-lit room. Silence distractions and make sure your household knows not to intrude. Wear professional attire and use appropriate body language. Check your internet connection and log in early. After your interview is done—reiterate your interest in the role and follow up with all interviews showing your appreciation for their time while expressing how interested you are. Good luck!

## INITIATIVES TO SUPPORT PD FOR DIVERSE WORKFORCE: WHAT WE CAN LEARN FROM INDUSTRY

*Emily Elswick*

*VP, Office of the CEO*

*Medtronic, Minneapolis, USA*

The picture inside of medical technology companies looks much different than it did in the early 2000s. Industry has made significant advances in ensuring the development of our employees with a focus on increasing our investment in and progress towards a more diverse workforce and one that reflects the customers and patients we serve around the globe. Whether it is diversity of thought, experience, ethnicity or gender, the advancements are visible and paying off.

Our employees are better equipped to manage the diverse stakeholders in healthcare, the ever-changing competitive landscape, the increase in innovation, the regulatory environment and the increased voice and needs of patients. It is no longer only about the physician and the effective use of a device or administering the therapy. Ensuring our businesses, regions and functions, understand the changing world and are educated to lean into that change is critical for the long-term success of the company. Industry has stood up programming to advance the knowledge and expertise needed to be a partner in healthcare today. Training our employees is critical. But it’s just as important to invest in and augment the support needed by health systems, governments, and societies to educate not only on the devices and therapies but the changing landscape of healthcare and the future of physicians and staff around the globe. Areas of focus; leading through change, optimizing for efficiencies, value-based healthcare, the use of AI in healthcare, developing practice or specialty programs, implementing new platform technologies and therapies into institutions, engaging with government and regulatory agencies, balancing work, and life, building a diverse, inclusive, and equitable work environment, and much more. The needs are great, and industry can help by ensuring the professional development of their employees and that of those we work to support. The more knowledgeable, and relevant leaders can be in this changing world of healthcare, the more impact we can make, together.

Also, sharing and gaining visibility and recognition for efforts that are making a difference are critical as well. Recently, Medtronic climbed to No. 2 on DiversityInc’s 2023 Top 50 Companies for Diversity. This is our highest-ever ranking. A commitment to inclusion, diversity and equity accelerates innovation, improves the well-being of our employees and creates more opportunities to bring our life-saving technologies to more patients around the world. These commitments go further into our organization as well and one of those impactful investments is the partnership to advance women thoracic surgeons as part of the ESTS Women in General Thoracic Surgery yearlong fellowship program. Educating, empowering and elevating women surgeons to improve their skills, work environment, personal and professional lives align to our mission, and we are proud to partner to increase the impact women in thoracic surgery can make and continue to break barriers and reach new heights for women in this critical specialty.

## SUPPORT FROM ALLIES

*Brendon M. Stiles, MD*

*Professor and Chief, Thoracic Surgery and Surgical Oncology*

*Albert Einstein College of Medicine and Montefiore Medical Center, New York, USA*

Developing a network of support for PD is critical for young surgeons, particularly for women or for those from underrepresented backgrounds. Although we are finally starting to see more women in leadership roles in our specialty, we must acknowledge that the majority of the thoracic surgery workforce are males. This perhaps makes it challenging for young female surgeons to find female mentors or sponsors at their local institution. Trainees and young surgeons should recognize however that they may often benefit from the perspective of non-thoracic surgeons. Identifying strong female leaders in any specialty or even in administrative positions is a great way to network and to develop a support system for PD and to gain perspective that extends outside the walls of thoracic surgery. Furthermore, young female surgeons should not hesitate to approach and utilize the experience of receptive male thoracic surgeons, as many are willing and capable advocates.

Among these allies who they do cultivate, female surgeons should identify mentors but should actively seek out sponsorship. Mentorship is the better understood of the 2 roles and generally consists of sharing knowledge, providing guidance and feedback and offering advice. Mentorship is often mutually beneficial to both the mentor and the mentee. As such, mentors are often relatively easy to identify. Sponsorship, however, can be seen as a three-way relationship in which sponsors help to manage *others*’ views of the sponsored surgeon and help to shape external opinions of their protégés. This is critical for pre- and post-interview relationship management. Although sponsorship supports and builds on mentorship, more risk is inherent for the sponsor as they must put their reputation and professional branding behind their protégé. Others have previously described the ABCDs of sponsorship to include Amplifying, Boosting, Connecting, and Defending the people that they sponsor [8]. For young female surgeons, sponsorship is likely even more important than mentorship. Female surgeons should therefore seek out sponsors, actively engage with them and use sponsorship to address the deep-seated challenges of PD in the thoracic surgery workforce.

## CONCLUSIONS

Clear priorities for actions to achieve a diverse workforce emerged in recent surveys and published reports in the field of thoracic surgery. ESTS and other organizations have committed to increase the availability of PD initiatives in most of their membership’s countries. Giving the young generations fair and unbiased resources to be ready for their careers’ journey would certainly be an important step in the context of a multilevel approach consisting of a combination of cultural and institutional methods that address the deeply rooted causes that lead to gender disparity in academic and clinical settings.

## References

[1] Pompili C , Opitz I, Backhus L, Leschber G, Veronesi G, Lauk O et al The impact of gender bias in cardiothoracic surgery in Europe: a European Society of Thoracic Surgeons and European Association for Cardio-Thoracic Surgery survey. Eur J Cardiothorac Surg 2022;61:1390–9. 35092281 10.1093/ejcts/ezac034 PMC9746891

[2] Parini S , Lucidi D, Azzolina D, Verdi D, Frigerio I, Gumbs AA et al Women in Surgery Italia: national survey assessing gender-related challenges. J Am Coll Surg 2021;233:583–92 e2. 34438082 10.1016/j.jamcollsurg.2021.08.675

[3] Erkmen CP , Ortmeyer KA, Pelletier GJ, Preventza O, Cooke DT; Society of Thoracic Surgeons Workforce on Diversity and Inclusion. An approach to diversity and inclusion in cardiothoracic surgery. Ann Thorac Surg 2021;111:747–52. 33345789 10.1016/j.athoracsur.2020.10.056 PMC8240968

[4] Olive JK , Preventza OA, Blackmon SH, Antonoff MB. Representation of women in the Society of Thoracic Surgeons authorship and leadership positions. Ann Thorac Surg 2020;109:1598–604. 31520640 10.1016/j.athoracsur.2019.07.069

[5] Yong V , Rostmeyer K, Deng M, Chin K, Graves EKM, Ma GX et al Gender differences in cardiothoracic surgery letters of recommendation. J Thorac Cardiovasc Surg 2023. 10.1016/j.jtcvs.2023.03.027. PMC10592592 37156362

[6] Bhama AR , Ritz EM, Anand RJ, Auyang ED, Lipman J, Greenberg JA et al Imposter syndrome in surgical trainees: clance imposter phenomenon scale assessment in general surgery residents. J Am Coll Surg 2021;233:633–8. 34384871 10.1016/j.jamcollsurg.2021.07.681

[7] Billan DR. The tallest poppy successful women pay a high price for success. 2023. https://insightplus.mja.com.au/2023/7/tall-poppies-navigating-gender-bias-as-a-woman-in-medicine/.

[8] Chow R. Don’t just mentor women and people of color. Sponsor them. *Harvard Business Review*, 2021.

